# Predictive signature of response to neoadjuvant chemotherapy in muscle-invasive bladder cancer integrating mRNA expression, taxonomic subtypes, and clinicopathological features

**DOI:** 10.3389/fonc.2023.1155244

**Published:** 2023-08-03

**Authors:** Albert Font, Montserrat Domenech, Jose Luis Ramirez, Miriam Marqués, Raquel Benítez, Vicenç Ruiz de Porras, José L. Gago, Cristina Carrato, Francesc Sant, Hector Lopez, Daniel Castellano, Nuria Malats, M. Luz Calle, Francisco X. Real

**Affiliations:** ^1^ Medical Oncology Department, Institut Català d’Oncologia, Hospital Universitari Germans Trias i Pujol, Badalona, Barcelona, Spain; ^2^ Badalona Applied Research Group in Oncology (B-ARGO), Germans Trias i Pujol Research Institute (IGTP), Badalona, Barcelona, Spain; ^3^ Medical Oncology Department, Althaia Xarxa Assistencial Universitària de Manresa, Manresa, Spain; ^4^ Hematology Service, Institut Català d'Oncologia (ICO) Badalona-Hospital Germans Trias i Pujol, Lymphoid Neoplasms Group, Josep Carreras Leukemia Research Institute (IJC), Badalona, Spain; ^5^ Epithelial Carcinogenesis Group, Spanish National Cancer Research Centre (CNIO) and CIBERONC, Madrid, Spain; ^6^ Genetic and Molecular Epidemiology Group, Spanish National Cancer Research Centre (CNIO), and CIBERONC, Madrid, Spain; ^7^ Urology Department, Hospital Universitari Germans Trias I Pujol, Badalona, Barcelona, Spain; ^8^ Pathology Department, Hospital Universitari Germans Trias I Pujol, Badalona, Barcelona, Spain; ^9^ Pathology Department, Althaia Xarxa Assistencial Universitària de Manresa, Manresa, Spain; ^10^ Urology Department, Althaia Xarxa Assistencial Universitària de Manresa, Manresa, Spain; ^11^ Medical Oncology Department, University Hospital 12 de Octubre, Madrid, Spain; ^12^ Biosciences Department, Faculty of Sciences, Technology, University of Vic-Central University of Catalonia, Vic, Barcelona, Spain; ^13^ Epithelial Carcinogenesis Group, Spanish National Cancer Research Centre (CNIO), Madrid, Spain; ^14^ Centre for Biomedical Research in Cancer Network (CIBERONC), Madrid, Spain; ^15^ Department of Medicine and Life Sciences, Universitat Pompeu Fabra, Barcelona, Spain

**Keywords:** bladder cancer, integrated signature, mRNA expression, neoadjuvant chemotherapy, pathological response, taxonomical classification

## Abstract

**Background and objective:**

Neoadjuvant chemotherapy (NAC) followed by cystectomy is the standard of care in muscle-invasive bladder cancer (MIBC). Pathological response has been associated with longer survival, but no currently available clinicopathological variables can identify patients likely to respond, highlighting the need for predictive biomarkers. We sought to identify a predictive signature of response to NAC integrating clinical score, taxonomic subtype, and gene expression.

**Material and methods:**

From 1994 to 2014, pre-treatment tumor samples were collected from MIBC patients (stage T2-4N0/+M0) at two Spanish hospitals. A clinical score was determined based on stage, hydronephrosis and histology. Taxonomic subtypes (BASQ, luminal, and mixed) were identified by immunohistochemistry. A custom set of 41 genes involved in DNA damage repair and immune response was analyzed in 84 patients with the NanoString nCounter platform. Genes related to pathological response were identified by LASSO penalized logistic regression. NAC consisted of cisplatin/methotrexate/vinblastine until 2000, after which most patients received cisplatin/gemcitabine. The capacity of the integrated signature to predict pathological response was assessed with AUC. Overall survival (OS) and disease-specific survival (DSS) were analyzed with the Kaplan-Meier method.

**Results:**

LASSO selected eight genes to be included in the signature (RAD51, IFNγ, CHEK1, CXCL9, c-MET, KRT14, HERC2, FOXA1). The highest predictive accuracy was observed with the inclusion in the model of only three genes (RAD51, IFNɣ, CHEK1). The integrated clinical-taxonomic-gene expression signature including these three genes had a higher predictive ability (AUC=0.71) than only clinical score plus taxonomic subtype (AUC=0.58) or clinical score alone (AUC=0.56). This integrated signature was also significantly associated with OS (p=0.02) and DSS (p=0.02).

**Conclusions:**

We have identified a predictive signature for response to NAC in MIBC patients that integrates the expression of three genes with clinicopathological characteristics and taxonomic subtypes. Prospective studies to validate these results are ongoing.

## Introduction

Neoadjuvant cisplatin-based chemotherapy followed by cystectomy is the standard of care for patients with muscle-invasive bladder cancer (MIBC), but the implementation of neoadjuvant chemotherapy (NAC) is relatively low in clinical practice (1), mainly due to a limited survival benefit. NAC-plus-cystectomy was associated with an overall improvement of only 5-8% in 5-year survival compared to cystectomy alone (2, 3). The benefit of NAC seems to be limited to patients with a pathological complete response (pCR) (4), whereas delaying cystectomy can negatively impact the prognosis of patients who do not respond to NAC and who could more effectively be treated with early cystectomy or alternative therapies. At present, however, there are no baseline clinicopathological variables that can identify patients likely to benefit from NAC, highlighting the clear yet unmet need for predictive markers of response.

Despite the urgent need for new active drugs for the treatment of bladder cancer, cisplatin-based regimens remain central to the multimodal treatment strategy for MIBC. The mechanism of action of cisplatin is based on the formation of DNA interstrand cross-links, and the analysis of genes involved in the different pathways of DNA damage repair (DDR) may help identify predictive biomarkers of response to cisplatin. In recent years, significant advances in the molecular characterization of bladder cancer have made it possible to identify new potential prognostic and predictive biomarkers, as well as new therapeutic targets (5). Several studies have reported that *ERCC2*, *ATM*, *RB1* and *FANCC* mutations are associated with response to cisplatin-based NAC (6–10). Recently, Gil-Jimenez et al. (11) have tried to validate these genomic biomarkers (ERCC2, ERBB2, ATM, RB1 and FANCC) in two retrospective cohorts of MIBC patients, but only deleterious mutations in ERCC2 were associated with pathological response to NAC. Likewise, some clinical trials, such as the RETAIN trial (NCT02710734), are evaluating the analysis of these biomarkers for use in selecting patients for bladder-sparing treatment based on clinical response to NAC and a favorable molecular profile. However, the prevalence of mutations in these genes in MIBC is relatively low (2-20%) (www.cbioportal.org), which limits their usefulness as predictive markers. Although other potential biomarkers of response have been proposed, none has yet been implemented in clinical practice (1, 12).

DDR is a complex process involving several DNA repair pathways, making it necessary to analyze more extensive panels of genes. MY Teo and colleagues reported the analysis of genomic alterations in a panel of 34 DDR genes in advanced bladder cancer and found alterations in at least one of these genes in 47% of patients; these alterations were associated with improved outcome (13). However, the potential role of a transcriptomic signature of DDR genes as a predictive biomarker of response to NAC has not been fully explored.

Gene RNA expression analysis has led to a general consensus on the identification of molecular subtypes in MIBC that are associated with specific clinicopathological characteristics, response to chemotherapy, and prognosis (14, 15). To date, however, studies have shown conflicting results regarding the capacity of molecular classification to predict response to NAC in MIBC. While some groups have reported that basal and non-luminal tumors are associated with better response to NAC (16, 17), other authors (18) have found that the basal/squamous (BASQ) subtype had the poorest response to NAC. Additionally, Sjödal et al. (19) and Kamoun et al. (15), found that tumors classified by the LundTax system as genomically unstable and urothelial-like tumors had a better response to NAC than those classified as BASQ tumors. Our group has recently proposed an immunohistochemistry (IHC)-based taxonomic subtyping model for MIBC patients treated with NAC. Using four markers (KRT5/6, KRT14, FOXA1 and GATA3), our model defines three subtypes of MIBC: BASQ, luminal, and mixed. Patients with BASQ tumors had a significantly higher probability of achieving a pCR to NAC than those with luminal or mixed tumors (20).

While our understanding of the MIBC molecular landscape is incomplete, integrative analyses can potentially enhance our knowledge of the biological processes involved in response to NAC. To explore this potential, we have analyzed data from 112 MIBC patients treated with NAC followed by cystectomy. We focused on a panel of 41 genes involved in DDR, immune response, molecular subtypes, and other cellular processes with the potential to provide predictive information. We then combined the gene expression results with the previously defined IHC-based taxonomic subtypes (20) and patient clinicopathological characteristics to generate an integrated predictive signature of response to NAC.

## Materials and methods

### Study design and patients

This was a retrospective study of MIBC patients treated with NAC between 1994 and 2014 at two Spanish hospitals (Institut Català d´Oncologia, Hospital Germans Trias i Pujol, Badalona; Fundació Althaia, Manresa, Barcelona). MIBC was identified by transurethral resection of the bladder tumor (TURBT). Patients clinically staged with thoracic and abdominal/pelvic computed tomography and classified as T2-4aN0-2M0 were candidates for NAC followed by cystectomy. All patients provided their signed informed consent and the study was approved by the institutional review boards of the two hospitals.

NAC was administered to 215 patients and consisted of cisplatin, methotrexate, and vinblastine (CMV) until 2000, after which most patients received cisplatin plus gemcitabine (CG) and 10% of patients received carboplatin plus gemcitabine (CaG). A total of 112 patients were included in the study. The remaining 103 patients were excluded because tumor tissue was unavailable or insufficient for the analysis of all four IHC markers. The main reason for lack of tissue availability was that one of the hospitals is a referral center and the TURBT had been performed elsewhere.

Patient data were collected through a retrospective review of clinical and pathological records.

### IHC analysis

The detailed methods and results of the IHC analysis of expression of: KRT5/6 (PRB-160P, Covance, 1/2000; 0.5 µg/mL), KRT14 (PRB-155P, Covance, 1/2000; 0.5 µg/mL), GATA3 (CM405 A, Biocare Medical, 1/300) and FOXA1 (ab170933, Abcam, 1/100; 10 µg/mL) in this patient series have been reported elsewhere (13). Tumors were classified in three subtypes according to the IHC results: BASQ-like (FOXA1/GATA3 low; KRT5/6/14 high), luminal-like (FOXA1/GATA3 high; KRT5/6/14 low), and mixed (FOXA1/GATA3 high; KRT5/6 high; KRT14 low).

### RNA isolation and gene expression analysis

H&E-stained sections of TURBT formalin-fixed paraffin-embedded (FFPE) samples were reviewed to identify areas enriched in tumor cells (>30%); consecutive 10-μm sections were used to macrodissect the selected areas and isolate RNA. Samples were sheared by sonication with a S2 Covaris instrument (Wobrun, MA). RNA was purified using the truXTRAC FFPE microTube RNA Kit (Covaris) following the manufacturer’s instructions, eluted in a final volume of 30 µl, and immediately quantified using the Qubit 3 Fluorometer (Thermo Fisher Scientific). Gene expression analysis was conducted using the NanoString nCounter platform (NanoString Technologies). Based on an extensive literature review, we selected a custom code set consisting of a panel of 41 genes involved in DNA repair and immune response pathways, molecular subtyping, or other relevant cellular processes (**Supplementary Table 1**). *ACTB, GAPDH, HPRT1*, and *LDHA* were used as endogenous controls.

Four transcripts analyzed (*PDL1, PD1, IFNɣ* and *FANCC*) were detected in fewer than 25% of samples We then analyzed the expression of *PDL1, PD1*, and *IFNg* by RT-qPCR in the same set of tumor samples. *FANCC* expression could not be detected even by RT-qPCR and was therefore excluded from the analysis. Briefly, after RNA extraction and purification, retrotranscription was performed with Moloney murine leukemia virus (MMLV) reverse transcriptase (Thermo Fisher Scientific). Template cDNA was amplified using commercial TaqMan gene expression assays and TaqMan Universal Master Mix (Applied Biosystems, Foster City, USA). Relative gene expression was quantified according to the comparative ΔCt method as previously described (21) using β-actin (ACTB) as the endogenous control.

The final mRNA signature did not include *FANCC* and was thus based on the expression of 40 genes.

### Statistical analyses

The raw NanoString data was normalized according to the manufacturer’s instructions, followed by a log-transformation to ensure normal distribution. Samples and genes with >75% of expression values lower than the negative control were filtered out. Gene expression heatmaps were generated to visualize possible patterns of expression.

pCR was defined as the absence of detectable tumor in the cystectomy specimen (pT0N0); pathological partial response (pPR) was defined as downstaging to non-MIBC (<pT2N0). All remaining patients were considered non-responders. Samples were classified as having a favorable (pCR) or unfavorable (pPR and non-responders) response to NAC, based on the linear predictive value of the model. Univariate logistic regression and box-and-whisker plots were used to analyze the association of each gene with pathological response. To identify the integrated signature to predict response to NAC, a conditional modeling approach integrating transcriptomic and clinical data was used (22). First, the clinical score was defined by implementing a stepwise regression strategy using the clinical and pathological variables that a previous study by our group (20) had found to have the highest capacity to predict pathological response: clinical stage, presence/absence of hydronephrosis, and histological type. The clinical score was then analyzed together with the previously defined IHC-based taxonomic subtype (BASQ vs luminal/mixed tumors) (20). Finally, this model was then analyzed together with the gene expression data using the LASSO (Least Absolute Shrinkage and Selection Operator) penalized regression model (23). To avoid overfitting, we performed repeated cross-validation analysis (70% train, 30% test, 100 repetitions). Genes and clinical score were ranked according to their selection in the 100 LASSO iterations. The mean area under the ROC curve (AUC) predictive score of the selected models was calculated using the test datasets. The contribution of each selected gene to the overall AUC was also assessed using the test datasets.

Overall survival (OS) was calculated from the date of TURBT to the date of last follow-up or death from any cause. Disease-specific survival (DSS) was calculated from the date of cystectomy to the date of last follow-up or death related to MIBC. Kaplan-Meier curves were drawn for OS and DSS according to two categories of the integrated signature and compared with the log-rank test for the different predictive models.

## Results

### Patient characteristics

A total of 215 patients were treated at the two participating centers, 112 of whom were included in the study. Informative results of the gene expression analysis were available in only 84 samples, and further analyses were performed only on these patients. There were no significant differences in baseline characteristics between the 215 patients treated at the two participating centers, the 84 patients included in the final analysis, and the remaining 131 patients (**Supplementary Table 2**). **Table 1** shows the baseline clinical and pathological characteristics of the 84 patients. Median age was 65 years (range, 41-80); 87% had pure urothelial carcinomas and the remaining 13% had mixed histology, with predominance of squamous differentiation. Clinical staging was T2-4N0M0 for 68 (80%) patients, while 16 (20%) had nodal involvement. NAC consisted of CG in 59% of patients, CMV in 25%, CaG in 11%, and other cisplatin-based regimens in 5%. Baseline patient characteristics were similar in both participating institutions except for the presence of lymphovascular invasion (p= 0.05) (**Supplementary Table 3**). Twenty-eight patients (33%) had a pCR. Importantly, no significant differences were observed in baseline patient characteristics between responders (pCR) and pPR or non-responder patients, except for the presence of variant histology, which was associated with a poor pathological response (**Supplementary Table 4**). The minimum follow-up of patients was four years. At the time of analysis, 50 patients (59%) had died – 34 (40%) due to bladder cancer progression and 16 due to other causes not related with bladder cancer.

**Table 1:** Baseline patient characteristics and treatment outcomes in 84 patients with muscle-invasive bladder cancer (MIBC) treated with neoadjuvant chemotherapy (NAC) plus cystectomy.

Characteristic	N	%
**No. Patients**	84	
Gender		
** Male**	78	93
** Female**	6	7
**Age, yrs – median (range)**	65.3 (41-80)	
Histology		
** Urothelial**	73	87
** Variant histology (squamous)**	11	13
** Variant histology (other)**	0	0
**Lymphovascular invasion**		
** Yes**	12	14
** No**	72	86
Clinical tumor stage (cTNM)		
** T2N0M0**	9	10
** T3-4N0M0**	59	70
** T1-4N+M0**	16	20
Hydronephrosis		
** Yes**	38	45
** No**	46	55
Prior non-MIBC		
** Yes**	14	17
** No**	70	83
NAC regimen		
** CG** ** CaG**	50 9	59 11
** CMV**	21	25
** DD-MVAC**	4	5
Pathological response		
** Complete**	28	33
** Partial/non-response**	56	67

CG, cisplatin plus gemcitabine; CaG, carboplatin plus gemcitabine; CMV, cisplatin, methotrexate, and vinblastine; DD-MVAC, dose-dense methotrexate, vinblastine, doxorubicin, and cisplatin.

### Integrated signature and outcome

**Figure 1:**
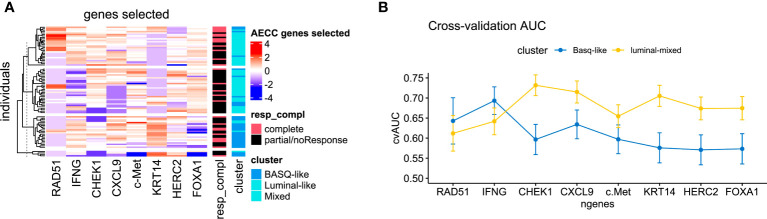
**(A)** Heat map of the expression of the eight genes selected by LASSO analysis stratified by taxonomic subtype and pathological response. **(B)** Predictive capacity of response to neoadjuvant chemotherapy of each of the selected genes in each taxonomic subtype (BASQ vs luminal/mixed tumors).

LASSO selected eight genes to be included in the training dataset for the predictive signature: *RAD51, IFNɣ, CHEK1, CXCL9, c-MET, KRT14, HERC2*, and *FOXA1* (**Figure 1**). **Table 2** lists the expression levels of these genes and their association with pathological response. We performed an integrated analysis combining the eight genes with the clinical score and the IHC-based taxonomic subtype to define the value of each of these variables separately and in combination in the final predictive model. The AUC for predictive accuracy for the model including only the clinical score was 0.56, which increased to 0.58 when the taxonomic subtype was added. When the model included the clinical score, the taxonomic subtype, and the eight genes, the AUC increased to 0.65 (**Table 3**).

**Table 2:** Genes selected by LASSO, showing their selection percentage and their mean and median log-expression.

			Mean		Median	
		Selection Percentage	pCR	pPR/no pR	pCR	pPR/no pR
1	***RAD51***	86	1.024	0.547	0	0
2	***IFNγ***	78	-0.974	-2.619	-0.224	-1.348
3	***CHEK1***	68	4.027	3.399	4.073	3.979
4	***CXCL9***	64	4.6	3.3	5.255	3.827
5	***c-Met***	44	4.403	4.577	4.494	4.715
6	***KRT14***	37	3.687	2.798	3.648	0
7	***HERC2***	32	5.526	5.894	5.619	5.833
8	***FOXA1***	27	5.521	6.112	6.384	6.753

pCR, pathological complete response; pPR, pathological partial response; pR, pathological response.

**Table 3:** Capacity of different models to predict pathological complete response: (1) clinical score alone, (2) clinical score plus IHC-based taxonomic subtype, (3) clinical score plus taxonomic subtype plus expression of eight LASSO-selected genes and (4) clinical score plus taxonomic subtype plus expression of three LASSO-selected genes.

Model	Variables included	LASSO-selected genes included	AUC
(1) Clinical score	cTNM Hydronephrosis Histological type	–	0.56
(2) Clinical score plus taxonomic subtype	cTNM Hydronephrosis Histological type BASQ vs luminal/mixed	–	0.58
(3) Clinical score plus taxonomic subtype plus gene expression	cTNM Hydronephrosis Histological type BASQ vs luminal/mixed Eight selected genes	*RAD51* *IFNγ* *CHEK1* *CXCL9* *c-Met* *KRT14* *HERC2* *FOXA1*	0.65
(4) Clinical score plus taxonomic subtype plus gene expression	cTNM Hydronephrosis Histological type BASQ vs luminal/mixed Three selected genes	*RAD51* *IFNγ* *CHEK1*	0.71

We then analyzed the impact of progressively adding each gene to the clinical-plus-taxonomic subtype model on the AUC of the integrated model. An increase in predictive accuracy was observed with the progressive inclusion in the model of the first three genes (*RAD51, IFNɣ, CHEK1*) – from an AUC of 0.56 to an AUC of 0.71 (**Table 3**). The remaining five genes did not significantly increase the predictive capacity of the model (**Table 4**), suggesting that it may well be sufficient to include only these three genes in the integrated signature and leading us to explore this possibility further.

**Table 4:** Predictive capacity of response to neoadjuvant chemotherapy by sequentially adding the selected genes to the clinical score plus taxonomic subtype, for all tumors and for each taxonomic subtype (luminal/mixed vs BASQ tumors).

	*RAD51*	*IFNγ*	*CHEK1*	*CXCL9*	*c-Met*	*KRT14*	*HERC2*	*FOXA1*
All tumors								
**cvAUC**	0.64	0.67	0.71	0.69	0.68	0.69	0.66	0.65
**95% CI**	0.63-0.67	0.66-0.71	0.69-0.73	0.67-0.72	0.66-0.70	0.66-0.71	0.64-0.68	0.62-0.67
Luminal/mixed								
**cvAUC**	0.61	0.64	0.73	0.71	0.65	0.71	0.67	0.67
**95% CI**	0.57-0.66	0.61-0.67	0.71-0.76	0.69-0.74	0.63-0.68	0.67-0.73	0.65-0.70	0.65-0.70
BASQ								
**cvAUC**	0.64	0.69	0.60	0.63	0.60	0.58	0.57	0.57
**95% CI**	0.58-0.70	0.66-0.73	0.56-0.63	0.60-0.67	0.56-0.63	0.54-0.61	0.53-0.61	0.53-0.61

cv, cross-validation.

When we analyzed the predictive capacity of the model including all eight genes for each of the two taxonomic subtypes (BASQ vs luminal/mixed tumors), the AUC was 0.67 (95% CI 0.65-0.70) for luminal/mixed tumors and 0.57 (95% CI 0.53-0.61) for BASQ tumors. In luminal/mixed tumors, the highest predictive capacity (AUC=0.73) was again obtained when including only the three genes (*RAD51*, *IFNγ*, *CHEK1*). In BASQ tumors, the highest predictive capacity (AUC=0.69) was obtained with a model including only two genes (*RAD51*, *IFNγ*) (**Table 4**; **Figure 1**).

**Figure 2:**
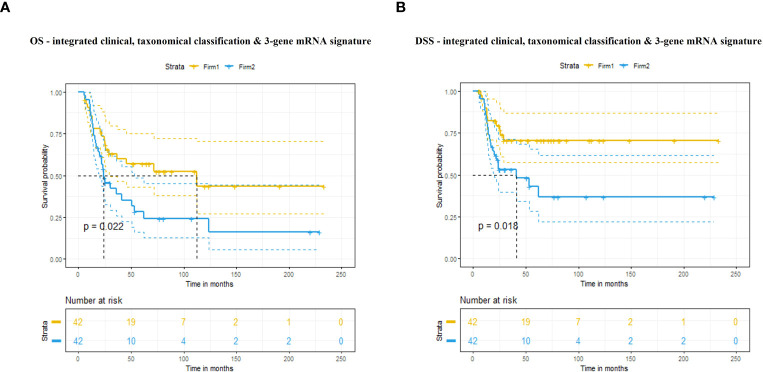
**(A)** Overall survival (OS) and **(B)** disease-specific survival (DSS) according to our predictive signature including clinical score, taxonomic subtype and expression of three genes.

The model including clinical score, taxonomic subtype and all eight genes was significantly associated with OS (p=0.008) and DSS (p=0.02). The model including clinical score, taxonomic subtype and only the three genes was also significantly associated with OS (p=0.02) and DSS (p=0.02) (**Figure 2**).

## Discussion

Quantitative methods of assessing tumor biology can provide information to improve the prediction of treatment response and outcome in bladder cancer patients (24, 25). Molecular signatures associated with homologous recombination deficiency in bladder cancer have been related to platinum sensitivity (26). Taber et al. (18) recently integrated genomics, transcriptomics, epigenomics and proteomics data with clinicopathological variables to define the probability of response to cisplatin-based chemotherapy in a cohort of bladder cancer patients treated with NAC or first-line chemotherapy for advanced or metastatic disease. Here we have described our integrated signature for predicting response to NAC in MIBC patients based on a clinical score (clinical stage, hydronephrosis, histology), IHC-based taxonomic subtype (BASQ vs luminal/mixed) (20), and mRNA expression of eight genes selected by LASSO from a panel of 40 genes. The eight genes include those related to DDR (*RAD51, CHEK1, HERC2*), signaling pathways (*c-MET*), surrogate luminal and basal markers (*FOXA1*, *KRT14*), and immune response (*IFNɣ*, *CXCL9*). The clinical score alone had a low capacity to predict pathological response (AUC=0.56) and this capacity increased slightly with the addition of the taxonomic subtype. However, when the eight genes were added to the model, the accuracy increased significantly (AUC=0.65). When we analyzed the impact of each of the eight genes, we found that *RAD51*, *IFNɣ* and *CHEK1* expression had the greatest impact on the predictive capacity of the integrated model (AUC=0.71). In fact, the predictive capacity did not increase when the remaining five genes were added. These results suggest that a model including only these three genes plus the clinical score and taxonomic subtype may predict response to NAC. This simplified model could facilitate its use in routine clinical practice.

Interestingly, two of the eight genes selected by LASSO are associated with immune response: *IFNɣ* and *CXCL9.* It is well known that platinum-based chemotherapy enhances antitumor immunomodulatory mechanisms. CXCL9 is a chemokine induced by IFNɣ that promotes T-cell recruitment in solid tumors (27). This chemokine activates the CXCR3 receptor, which has been associated with response to NAC (28). Previous studies have reported that tumor inflammation by upregulation of these genes is a prognostic factor in MIBC regardless of tumor subtype (29, 30). In fact, in the present study, *IFNɣ* mRNA expression was one of the three genes highly associated with response to NAC both in all patients and in the luminal/mixed and BASQ taxonomic subtypes.

Our signature also includes *RAD51* and *CHEK1*, both of which play a significant role in DDR. *RAD51* is involved in the activation of the homologous recombination pathway and double-strand break repair (31), and cytoplasmatic *RAD51* is involved in maintenance of the mitochondrial genome (32). Pataer et al. found a significant association between *RAD51* expression and survival in lung cancer patients treated with NAC (33). CHEK1, a serine/threonine-specific protein kinase, is required for checkpoint-mediated cell cycle arrest in response to DNA damage. Knockdown of *CHEK1* led to decreased cell viability and increased cisplatin sensitivity, and high *CHEK1* mRNA expression was associated with poor prognosis in several tumors, including bladder cancer (34, 35). In the present study, we have found that the predictive capacity of *CHEK1* was more pronounced in patients with luminal/mixed tumors than in those with BASQ tumors. In line with these findings, a recent study demonstrated the key role of *CHEK1* as a prognostic biomarker in luminal breast cancer patients (36).

Our study has several limitations, including its retrospective design and the inclusion of a relatively heterogenous group of patients, some of whom had regional lymph node involvement and some who were treated with carboplatin rather than cisplatin. However, we did not observe significant differences in pathological response according to the NAC regimen. In fact, three of nine patients (33%) achieved a pCR after carboplatin-based NAC, which is within the range obtained with cisplatin-based NAC. Likewise, pathological downstaging was observed in 50% of 16 patients with regional lymph node involvement, including 25% of pCR, suggesting that clinical stage is more a prognostic than a predictive factor of response to NAC. Furthermore, we have obtained informative results in only about 40% of the original cohort of 215 patients, primarily due to the lack of tumor tissue in about 25% of patients, in whom TURBT had not been performed in the participating hospitals. In addition, a large percentage of tumor samples had been obtained more than 20 years previously, which could have affected the quality of the molecular analysis. However, there were no significant differences in baseline characteristics between the 131 patients not included in the final analyses and the 84 patients with informative results. Although we are aware that the number of patients included could be a limitation of our study, a robust internal validation (100-fold cross-validation) showed our results to be sound. Finally, the expression of three genes was analyzed with both NanoString and RT-qPCR, which could have skewed the results. Despite these limitations, however, our results are promising and warrant validation in a prospective study.

Our model integrating the expression of three genes (*RAD51*, *IFNɣ*, *CHEK1*) with taxonomic subtypes and patient clinicopathological characteristics could help predict response to NAC in MIBC patients and could be easily incorporated into clinical practice. The identification of patients who can benefit from NAC may make it possible to implement bladder-sparing strategies, as in the RETAIN trial (NCT02710734). In contrast, patients identified as being potentially refractory to NAC should be offered alternative therapies, such as immediate cystectomy or neoadjuvant immunotherapy, which has shown promising results both alone and in combination with chemotherapy (37–39). Prospective studies to validate our results are ongoing.

## Data availability statement

The NanoString raw data used in this study have already been deposited in the GEO repository. GEO ID: GSE235067.

## Ethics statement

The studies involving human participants were reviewed and approved by Comitè d’Etica de la Investigació amb medicaments Hospital Universitari Germans Trias i Pujol. The patients/participants provided their written informed consent to participate in this study.

## Author contributions

Conception and design: AF, MD, JR, MM, NM, MC, FR. Acquisition of data: AF, MD, JG, HL, CC, FS. Analysis and interpretation of data: AF, MD, JR, VP, NM, MC, FR. Drafting of the manuscript: AF, MD, JR, NM, MC, FR. Critical revision of the manuscript: JR, JG, DC, NM, FR. Statistical analysis: RB, NM, MC. Obtaining funding: FR, NM, DC, AF. Supervision: AF, FR. All authors contributed to the article and approved the submitted version.
