# Supplementary material for: Predictive signature of response to neoadjuvant chemotherapy in muscle-invasive bladder cancer integrating mRNA expression, taxonomic subtypes, and clinicopathological features
Source: Front Oncol. 2023 Aug 3;13:1155244. doi: 10.3389/fonc.2023.1155244 (PMC10426739; doi:10.3389/fonc.2023.1155244)
Supplement: Supplementary file 1 [file Table_1.docx]

Supplementary Material

Predictive signature of response to neoadjuvant chemotherapy in muscle-invasive bladder cancer integrating mRNA expression, taxonomic subtypes, and clinicopathological features

**Albert Font*, Montserrat Domenech, Jose Luis Ramirez, Miriam Marqués, Raquel Benítez, José L. Gago, Cristina Carrato, Francesc Sant, Vicenç Ruiz de Porras, Hector Lopez, Daniel Castellano, Nuria Malats, M. Luz Calle, Francisco X. Real**

*** Correspondence:** Albert Font; afont@iconcologia.net

# Supplementary Tables

**Supplementary Table 1.** The 41 genes involved in DNA repair, molecular subtyping, immune response, or other cellular processes that were analyzed with the NanoString nCounter platform

| **DNA Repair** | | | | **Molecular Subtyping** | **Immune Response** | **Other Cellular Processes** |
| --- | --- | --- | --- | --- | --- | --- |
| *BRCA1* | *RAD51* | *PALB2* | *REV7*  *(MAD2L2)* | *KRT 5/6* | *IFNγ** | *c-Met* |
| *BRCA2* | *RAD50* | *CHEK1* | *POLθ* | *KRT14* | *LAG3* | *Axl* |
| *ERCC1* | *PARP* | *CHEK2* | *PTIP* | *GATA3* | *PD1** | *RON* |
| *ERCC2* | *Rif1* | *NBN* | *RNF8* | *FOXA1* | *PDL1** | *TERT* |
| *ERCC5* | *53BP1* | *FANCC** | *RNF168* |  | *CXCL9* |  |
| *ERCC6* | *ATM* | *FANCA* | *HERC2* |  |  |  |
| *Ku80* | *ATR* | *FANCD2* | *eIF3a* |  |  |  |

**PDL1, PD1, IFNɣ* and *FANCC* were detected in fewer than 25% of samples and excluded from further analysis by NanoString. The expression of *PDL1, PD1* and *IFNɣ* was subsequently analyzed by RT-qPCR.

**Supplementary Table 2.** Characteristics of 215 patients with muscle-invasive bladder cancer treated at the two participating centers, 131 patients not included in the final analyses, and 84 patients included in the final analyses.

| **Characteristics** | **Patients Treated**  **N=215**  **N (%)** | **Patients Excluded**  **N=131***  **N (%)** | **Patients Included**  **N=84**  **N (%)** | **p** |
| --- | --- | --- | --- | --- |
| **Sex** |  |  |  | 0.1 |
| Men | 199 (92.6) | 121 (92.4) | 78 (93.0) |  |
| Women | 16 (7.44) | 10 (7.6) | 6 (7.0) |  |
| **Age, yrs – median (range)** | 66 (59-71.5)] | 65 (58-70.5) | 65.3 (48-79) |  |
| **Histology** |  |  |  | 0.6137 |
| Urothelial | 183 (85.1) | 110 (84.1) | 73 (87.0) |  |
| Variant histology (squamous) | 23 (10.7) | 12 (9.1) | 11 (13.0) |  |
| Other | 9 (5.2) | 9 (6.8) | ---- |  |
| **Lymphovascular invasion** |  |  |  | 0.346 |
| No | 191 (87.4) | 119 (90.9) | 72 (85.7) |  |
| Yes | 24 (12.6) | 12 (9.1) | 12 (14.3) |  |
| **cTNM** |  |  |  | 0.3359 |
| T2N0M0 | 16 (7.9) | 7 (5.3) | 9 (10.7) |  |
| T3-4N0M0 | 158 (73.0) | 99 (75.5) | 59 (70.2) |  |
| T1-4N+M0 | 41 (19.1) | 25 (19.2) | 16 (19.1) |  |
| **Pathologic response** |  |  |  | 0.286 |
| Not assessable | 13 (6.0) | 13 (9.9) | --- |  |
| Complete response | 58 (27.0) | 30 (22.9) | 28 (33.3) |  |
| Partial / non-response | 144 (67.0) | 88 (67.2) | 56 (66.7) |  |
| **NAC regimen** |  |  |  | 0.1476 |
| CMV | 66 (30.7) | 45 (34.5) | 21 (25.0) |  |
| CG | 124 (57.7) | 74 (56.4) | 50 (59.5) |  |
| CaG | 20 (9.3) | 11 (8.4) | 9 (10.7) |  |
| DD-MVAC | 5 (2.3) | 1 (0.7) | 4 (4.8) |  |

NAC, neoadjuvant chemotherapy; CMV, cisplatin, methotrexate, and vinblastine; CG, cisplatin plus gemcitabine; CaG, carboplatin plus gemcitabine; DD-MVAC, dose-dense methotrexate, vinblastine, doxorubicin, and cisplatin

* Of the 215 patients treated at the two centers, 103 were excluded from the study because tumor tissue was unavailable or insufficient for the analysis of all four IHC markers. A further 28 patients were not included in the final analyses because gene expression results were not informative.

**Supplementary Table 3.** Baseline patient characteristics according to participating center

| **Patient characteristic** | **Center 1 (Badalona)**  **(n=51 p)** | **Center 2 (Manresa)**  **(n=33 p)** | **P** |
| --- | --- | --- | --- |
| **Age, years**  < 65  >65 | 24  27 | 12  21 | 0.4 |
| **Sex**  Male  Female | 48  3 | 30  3 | 0.7 |
| **Histology**  Urothelial  Variant histology | 44  7 | 26  7 | 0.4 |
| **Lymphovascular invasion**  Yes  No | 4  47 | 8  25 | 0.05 |
| **Clinical tumor stage (cTNM)**  T2-4N0M0  T2-4N+M0 | 43  8 | 26  7 | 0.4 |
| **Hydronephrosis**  Yes  No | 23  28 | 15  18 | 1 |
| **Prior non-MIBC**  Yes  No | 8  43 | 6  27 | 0.77 |
| **Chemotherapy regimen**  Cisplatin-based  Carboplatin-based (CaG) | 47  4 | 28  5 | 0.3 |

**Supplementary Table 4. Baseline patient characteristics according to pathological response**

| **Patient characterisitcs** | **Responders (n=28)** | **Non-responders (n= 56p)** | **P** |
| --- | --- | --- | --- |
| **Age, median**  < 65  >65 | 9  19 | 27  29 | 0.2 |
| **Sex**  Male  Female | 27  1 | 51  5 | 0.7 |
| **Histology**  Urothelial  Variant histology | 27  1 | 43  13 | 0.03 |
| **Lymphovascular invasion**  Yes  No | 3  25 | 9  47 | 0.74 |
| **Clinical tumor stage** (cTNM)  T2-4N0M0  T2-4N+M0 | 23  5 | 46  10 | 0.8 |
| **Hydronephrosis**  Yes  No | 8  20 | 30  26 | 0.2 |
| **Prior non-MIBC**  Yes  No | 4  24 | 10  46 | 0.76 |
| **Chemotherapy regimen**  Cisplatin-based  Carboplatin-based (CaG) | 25  3 | 50  6 | 1 |

Responders: pT0N0M0; Non-responders: pT1/Tis/T2-4 and/or pN+ ; NAC: neoadjuvant chemotherapy
